# *rac*-(3*S*,4*Z*)-3-Chloro-4-[2-(3-fluoro­benzyl­idene)hydrazinyl­idene]-1-methyl-3,4-dihydro-1*H*-2λ^6^,1-benzothia­zine-2,2-dione

**DOI:** 10.1107/S1600536812021654

**Published:** 2012-05-19

**Authors:** Muhammad Shafiq, M. Nawaz Tahir, Islam Ullah Khan, Saeed Ahmad

**Affiliations:** a Department of Chemistry, Government College University, Faisalabad, Pakistan; b University of Sargodha, Department of Physics, Sargodha, Pakistan; c Materials Chemistry Laboratory, Department of Chemistry, Government College University, Lahore, Pakistan; d Department of Chemistry, Gomal University, Dera Ismail Khan, K.P.K, Pakistan

## Abstract

In the title compound, C_16_H_13_ClFN_3_O_2_S, the dihedral angle between the benzene rings is 4.47 (3)°. The conformation of the thia­zine ring is a half-chair and the Cl atom is in an axial orientation. In the crystal, mol­ecules are linked by C—H⋯F inter­actions, generating *C*(12) chains propagating in [011]. Aromatic π–π stacking inter­actions [centroid–centroid separations = 3.753 (2) and 3.758 (2) Å] also occur.

## Related literature

For a related structure and background references, see: Shafiq *et al.* (2012 ▶). For further synthetic details, see: Shafiq *et al.* (2011**a* ▶,b* ▶). For ring conformations, see: Cremer & Pople (1975 ▶).
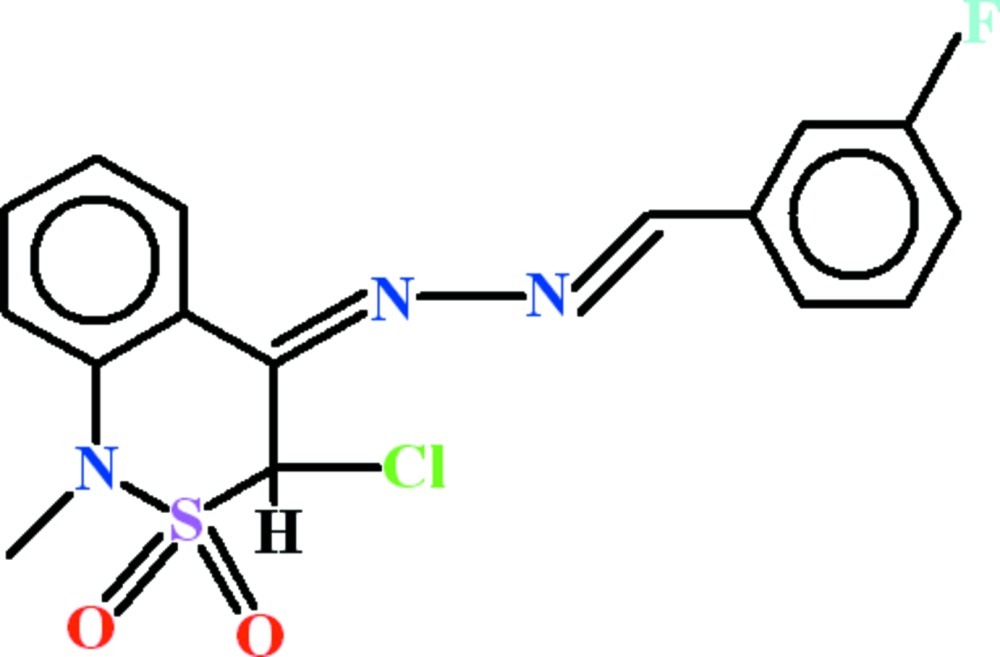


## Experimental

### 

#### Crystal data

C_16_H_13_ClFN_3_O_2_S
*M* *_r_* = 365.80
Triclinic, 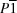

*a* = 7.0072 (3) Å
*b* = 8.9402 (4) Å
*c* = 13.3438 (6) Å
α = 98.184 (3)°
β = 90.510 (2)°
γ = 98.389 (3)°
*V* = 818.19 (6) Å^3^
*Z* = 2
Mo *K*α radiation
μ = 0.39 mm^−1^
*T* = 296 K
0.26 × 0.18 × 0.12 mm

#### Data collection

Bruker Kappa APEXII CCD diffractometer
Absorption correction: multi-scan (*SADABS*; Bruker, 2005 ▶) *T*_min_ = 0.930, *T*_max_ = 0.960
11874 measured reflections
2941 independent reflections
1744 reflections with *I* > 2σ(*I*)
*R*_int_ = 0.065

#### Refinement

*R*[*F* ^2^ > 2σ(*F* ^2^)] = 0.050
*wR*(*F* ^2^) = 0.120
*S* = 1.00
2941 reflections
218 parameters
H-atom parameters constrained
Δρ_max_ = 0.33 e Å^−3^
Δρ_min_ = −0.32 e Å^−3^

### 

Data collection: *APEX2* (Bruker, 2009 ▶); cell refinement: *SAINT* (Bruker, 2009 ▶); data reduction: *SAINT*; program(s) used to solve structure: *SHELXS97* (Sheldrick, 2008 ▶); program(s) used to refine structure: *SHELXL97* (Sheldrick, 2008 ▶); molecular graphics: *ORTEP-3 for Windows* (Farrugia, 1997 ▶) and *PLATON* (Spek, 2009 ▶); software used to prepare material for publication: *WinGX* (Farrugia, 1999 ▶) and *PLATON*.

## Supplementary Material

Crystal structure: contains datablock(s) global, I. DOI: 10.1107/S1600536812021654/hb6778sup1.cif

Structure factors: contains datablock(s) I. DOI: 10.1107/S1600536812021654/hb6778Isup2.hkl

Supplementary material file. DOI: 10.1107/S1600536812021654/hb6778Isup3.cml

Additional supplementary materials:  crystallographic information; 3D view; checkCIF report

## Figures and Tables

**Table 1:** Hydrogen-bond geometry (Å, °)

*D*—H⋯*A*	*D*—H	H⋯*A*	*D*⋯*A*	*D*—H⋯*A*
C5—H5⋯F1^i^	0.93	2.53	3.442 (5)	167

Symmetry code: (i) 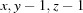.

**Fig. 1.**
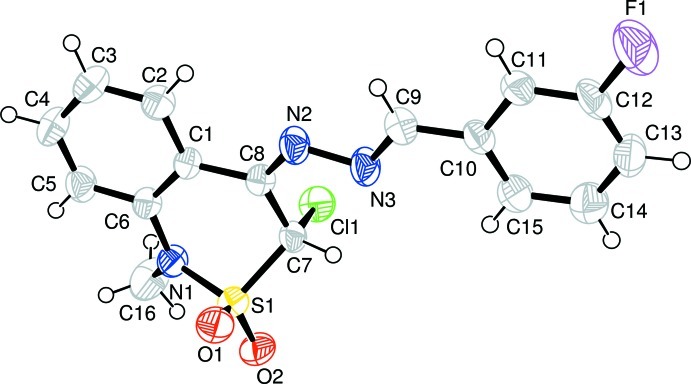
View of the title compound with displacement ellipsoids drawn at the 50% probability level.

**Fig. 2.**
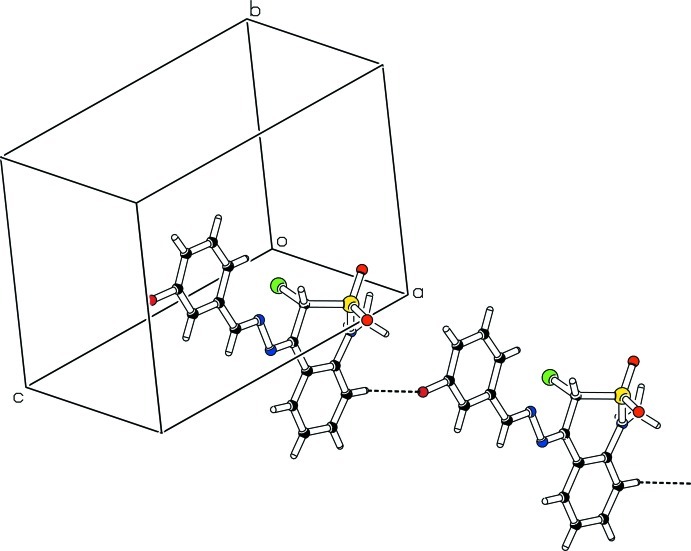
Partial packing diagram showing polymeric chains.

## supplementary crystallographic information

### Comment

As part of our ongoing synthetic and structural studies of thiazine derivatives (Shafiq *et al.*, 2012), we now describe the title compound, (I), (Fig. 1).

In (I), the benzene rings A (C1—C6) and B (C10—C15) are planar with r. m. s. deviation of 0.0040 and 0.0012 Å, respectively. The dihedral angle between A/B is 4.47 (3)°. The central group C (N2/N3/C9) is of course planar. The dihedral angle between A/C and B/C is 5.87 (7) and 1.48 (8)°, respectively. The thiazine ring D (C1/C6/N1/S1/C7/C8) is in the half-chair form, with the maximum puckering amplitude (Cremer & Pople, 1975), Q = 0.563 (3) Å. In the crystal, the molecules form chains due to H-bonding of C—H···F type (Table 1, Fig. 2). There exist π–π interactions between CgA···CgB^i^ [i = 1 - *x*, -*y*, 1 - *z*] and CgB···CgA^ii^ [ii = 2 - *x*, -*y*, 1 - *z*] at a distance of 3.758 (2) and 3.753 (2) Å, where CgA and CgB are the centroids of benzene rings A and B, respectively.

### Experimental

The Schiff base derivative of (4*Z*)-4-hydrazinylidene-1-methyl-3,4-dihydro -1*H*-2,1-benzothiazine 2,2-dioxide and 3-flourobenzaldehyde was prepared using the method reported previously (Shafiq *et al.* 2011*b*). The chlorination of the schiff base was undertaken using *N*-chloro succinimide and dibenzoylperoxide (Shafiq *et al.*, 2011*a*). The crude product of (I) was re-crystallized in ethyl acetate to obtain yellow needles of the title compound.

### Refinement

The H-atoms were positioned geometrically (C–H = 0.93–0.96 Å) and refined as riding with *U*_iso_(H) = *xU*_eq_(C), where *x* = 1.5 for methyl and *x* = 1.2 for aryl H-atoms.

### Crystal data

**Table:**

C_16_H_13_ClFN_3_O_2_S	*Z* = 2
*M**_r_* = 365.80	*F*(000) = 376
Triclinic, *P*1	*D*_x_ = 1.485 Mg m^−^^3^
Hall symbol: -P 1	Mo *K*α radiation, λ = 0.71073 Å
*a* = 7.0072 (3) Å	Cell parameters from 1744 reflections
*b* = 8.9402 (4) Å	θ = 2.3–25.3°
*c* = 13.3438 (6) Å	µ = 0.39 mm^−^^1^
α = 98.184 (3)°	*T* = 296 K
β = 90.510 (2)°	Needle, yellow
γ = 98.389 (3)°	0.26 × 0.18 × 0.12 mm
*V* = 818.19 (6) Å^3^	

### Data collection

**Table:**

Bruker Kappa APEXII CCD diffractometer	2941 independent reflections
Radiation source: fine-focus sealed tube	1744 reflections with *I* > 2σ(*I*)
Graphite monochromator	*R*_int_ = 0.065
Detector resolution: 8.10 pixels mm^-1^	θ_max_ = 25.3°, θ_min_ = 2.3°
ω scans	*h* = −8→8
Absorption correction: multi-scan (*SADABS*; Bruker, 2005)	*k* = −10→10
*T*_min_ = 0.930, *T*_max_ = 0.960	*l* = −16→16
11874 measured reflections	

### Refinement

**Table:**

Refinement on *F*^2^	Primary atom site location: structure-invariant direct methods
Least-squares matrix: full	Secondary atom site location: difference Fourier map
*R*[*F*^2^ > 2σ(*F*^2^)] = 0.050	Hydrogen site location: inferred from neighbouring sites
*wR*(*F*^2^) = 0.120	H-atom parameters constrained
*S* = 1.00	*w* = 1/[σ^2^(*F*_o_^2^) + (0.0478*P*)^2^ + 0.0889*P*] where *P* = (*F*_o_^2^ + 2*F*_c_^2^)/3
2941 reflections	(Δ/σ)_max_ < 0.001
218 parameters	Δρ_max_ = 0.33 e Å^−^^3^
0 restraints	Δρ_min_ = −0.32 e Å^−^^3^

### Special details

**Table:**

Geometry. Bond distances, angles *etc*. have been calculated using the rounded fractional coordinates. All su's are estimated from the variances of the (full) variance-covariance matrix. The cell e.s.d.'s are taken into account in the estimation of distances, angles and torsion angles
Refinement. Refinement of *F*^2^ against ALL reflections. The weighted *R*-factor *wR* and goodness of fit *S* are based on *F*^2^, conventional *R*-factors *R* are based on *F*, with *F* set to zero for negative *F*^2^. The threshold expression of *F*^2^ > σ(*F*^2^) is used only for calculating *R*-factors(gt) *etc*. and is not relevant to the choice of reflections for refinement. *R*-factors based on *F*^2^ are statistically about twice as large as those based on *F*, and *R*- factors based on ALL data will be even larger.

### Fractional atomic coordinates and isotropic or equivalent isotropic displacement parameters (Å^2^)

**Table:**

	*x*	*y*	*z*	*U*_iso_*/*U*_eq_	
Cl1	0.49246 (13)	0.08066 (10)	0.23725 (7)	0.0532 (4)	
S1	0.86510 (13)	0.02559 (10)	0.15593 (7)	0.0428 (3)	
F1	0.8027 (4)	0.4684 (3)	0.88468 (18)	0.0894 (11)	
O1	1.0538 (3)	0.0163 (3)	0.19335 (18)	0.0532 (9)	
O2	0.8416 (4)	0.1283 (3)	0.08580 (18)	0.0561 (10)	
N1	0.7533 (4)	−0.1425 (3)	0.1110 (2)	0.0426 (10)	
N2	0.7629 (4)	−0.0292 (3)	0.4293 (2)	0.0459 (11)	
N3	0.7744 (4)	0.1263 (3)	0.4676 (2)	0.0483 (11)	
C1	0.7272 (4)	−0.2167 (3)	0.2815 (3)	0.0336 (11)	
C2	0.7020 (5)	−0.3359 (4)	0.3404 (3)	0.0450 (12)	
C3	0.6839 (5)	−0.4857 (4)	0.2968 (3)	0.0516 (16)	
C4	0.6917 (5)	−0.5215 (4)	0.1934 (3)	0.0523 (16)	
C5	0.7157 (5)	−0.4082 (4)	0.1340 (3)	0.0478 (12)	
C6	0.7322 (4)	−0.2558 (4)	0.1761 (3)	0.0365 (12)	
C7	0.7362 (4)	0.0683 (4)	0.2675 (2)	0.0369 (12)	
C8	0.7431 (4)	−0.0574 (3)	0.3326 (3)	0.0349 (11)	
C9	0.7800 (5)	0.1472 (4)	0.5641 (3)	0.0460 (14)	
C10	0.7926 (5)	0.2996 (4)	0.6232 (3)	0.0403 (12)	
C11	0.7939 (5)	0.3134 (4)	0.7280 (3)	0.0446 (12)	
C12	0.8044 (5)	0.4569 (5)	0.7816 (3)	0.0509 (14)	
C13	0.8143 (5)	0.5861 (4)	0.7388 (3)	0.0555 (16)	
C14	0.8139 (6)	0.5733 (5)	0.6347 (3)	0.0616 (17)	
C15	0.8029 (5)	0.4312 (4)	0.5773 (3)	0.0520 (16)	
C16	0.6640 (7)	−0.1733 (4)	0.0094 (3)	0.0738 (19)	
H2	0.69751	−0.31273	0.41045	0.0537*	
H3	0.66630	−0.56307	0.33701	0.0617*	
H4	0.68056	−0.62314	0.16378	0.0630*	
H5	0.72107	−0.43369	0.06412	0.0575*	
H7	0.79712	0.16603	0.30513	0.0445*	
H9	0.77593	0.06299	0.59809	0.0550*	
H11	0.78773	0.22787	0.76088	0.0531*	
H13	0.82112	0.68101	0.77869	0.0670*	
H14	0.82110	0.66031	0.60328	0.0737*	
H15	0.80241	0.42290	0.50700	0.0627*	
H16A	0.73119	−0.24304	−0.03305	0.1104*	
H16B	0.67065	−0.07948	−0.01841	0.1104*	
H16C	0.53138	−0.21768	0.01300	0.1104*	

### Atomic displacement parameters (Å^2^)

**Table:**

	*U*^11^	*U*^22^	*U*^33^	*U*^12^	*U*^13^	*U*^23^
Cl1	0.0468 (6)	0.0544 (6)	0.0638 (7)	0.0169 (5)	0.0014 (5)	0.0171 (5)
S1	0.0479 (6)	0.0375 (6)	0.0431 (6)	0.0047 (4)	0.0054 (4)	0.0072 (4)
F1	0.118 (2)	0.096 (2)	0.0487 (17)	0.0188 (16)	0.0047 (15)	−0.0117 (13)
O1	0.0366 (14)	0.0522 (16)	0.0688 (18)	0.0026 (12)	0.0021 (13)	0.0062 (14)
O2	0.0778 (19)	0.0452 (16)	0.0494 (17)	0.0094 (13)	0.0105 (14)	0.0197 (13)
N1	0.0589 (19)	0.0380 (18)	0.0290 (17)	0.0038 (14)	−0.0007 (14)	0.0021 (14)
N2	0.062 (2)	0.0408 (19)	0.0339 (19)	0.0118 (15)	−0.0019 (15)	−0.0016 (14)
N3	0.070 (2)	0.0406 (19)	0.0321 (19)	0.0104 (16)	−0.0009 (16)	−0.0043 (14)
C1	0.0342 (19)	0.031 (2)	0.035 (2)	0.0057 (15)	−0.0041 (16)	0.0025 (16)
C2	0.049 (2)	0.044 (2)	0.042 (2)	0.0071 (18)	−0.0016 (18)	0.0060 (19)
C3	0.059 (3)	0.038 (2)	0.059 (3)	0.0051 (19)	−0.007 (2)	0.014 (2)
C4	0.064 (3)	0.032 (2)	0.059 (3)	0.0044 (19)	−0.007 (2)	0.004 (2)
C5	0.057 (2)	0.044 (2)	0.040 (2)	0.0084 (19)	0.0006 (19)	−0.0031 (19)
C6	0.038 (2)	0.036 (2)	0.035 (2)	0.0062 (16)	−0.0027 (16)	0.0032 (17)
C7	0.040 (2)	0.034 (2)	0.034 (2)	0.0038 (16)	0.0010 (16)	−0.0024 (16)
C8	0.0325 (19)	0.035 (2)	0.036 (2)	0.0037 (15)	−0.0034 (16)	0.0025 (16)
C9	0.048 (2)	0.047 (2)	0.043 (3)	0.0120 (18)	0.0026 (19)	0.0019 (19)
C10	0.041 (2)	0.046 (2)	0.032 (2)	0.0069 (17)	0.0038 (16)	−0.0009 (18)
C11	0.049 (2)	0.047 (2)	0.036 (2)	0.0043 (18)	0.0045 (18)	0.0031 (18)
C12	0.054 (2)	0.066 (3)	0.027 (2)	0.006 (2)	0.0025 (18)	−0.010 (2)
C13	0.055 (3)	0.042 (2)	0.065 (3)	0.007 (2)	0.006 (2)	−0.008 (2)
C14	0.071 (3)	0.056 (3)	0.056 (3)	0.007 (2)	0.003 (2)	0.005 (2)
C15	0.062 (3)	0.053 (3)	0.039 (2)	0.006 (2)	0.0028 (19)	0.002 (2)
C16	0.123 (4)	0.056 (3)	0.039 (3)	0.002 (3)	−0.016 (3)	0.008 (2)

### Geometric parameters (Å, º)

**Table:**

Cl1—C7	1.774 (3)	C10—C11	1.386 (6)
S1—O1	1.427 (2)	C10—C15	1.395 (5)
S1—O2	1.426 (3)	C11—C12	1.369 (6)
S1—N1	1.620 (3)	C12—C13	1.353 (6)
S1—C7	1.772 (3)	C13—C14	1.377 (6)
F1—C12	1.365 (5)	C14—C15	1.378 (6)
N1—C6	1.418 (5)	C2—H2	0.9300
N1—C16	1.461 (5)	C3—H3	0.9300
N2—N3	1.402 (4)	C4—H4	0.9300
N2—C8	1.281 (5)	C5—H5	0.9300
N3—C9	1.274 (5)	C7—H7	0.9800
C1—C2	1.404 (5)	C9—H9	0.9300
C1—C6	1.402 (6)	C11—H11	0.9300
C1—C8	1.477 (4)	C13—H13	0.9300
C2—C3	1.370 (5)	C14—H14	0.9300
C3—C4	1.375 (6)	C15—H15	0.9300
C4—C5	1.365 (5)	C16—H16A	0.9600
C5—C6	1.386 (5)	C16—H16B	0.9600
C7—C8	1.521 (5)	C16—H16C	0.9600
C9—C10	1.464 (5)		
			
O1—S1—O2	119.60 (17)	F1—C12—C13	118.7 (4)
O1—S1—N1	110.77 (16)	C11—C12—C13	124.2 (4)
O1—S1—C7	103.50 (14)	C12—C13—C14	118.3 (4)
O2—S1—N1	108.96 (15)	C13—C14—C15	119.8 (4)
O2—S1—C7	111.18 (16)	C10—C15—C14	120.9 (4)
N1—S1—C7	101.18 (15)	C1—C2—H2	119.00
S1—N1—C6	117.9 (2)	C3—C2—H2	119.00
S1—N1—C16	121.1 (2)	C2—C3—H3	120.00
C6—N1—C16	120.9 (3)	C4—C3—H3	120.00
N3—N2—C8	113.5 (3)	C3—C4—H4	120.00
N2—N3—C9	111.1 (3)	C5—C4—H4	120.00
C2—C1—C6	117.8 (3)	C4—C5—H5	119.00
C2—C1—C8	118.9 (3)	C6—C5—H5	119.00
C6—C1—C8	123.2 (3)	Cl1—C7—H7	109.00
C1—C2—C3	121.3 (4)	S1—C7—H7	109.00
C2—C3—C4	119.9 (3)	C8—C7—H7	109.00
C3—C4—C5	120.2 (3)	N3—C9—H9	119.00
C4—C5—C6	121.1 (4)	C10—C9—H9	119.00
N1—C6—C1	121.4 (3)	C10—C11—H11	121.00
N1—C6—C5	118.9 (3)	C12—C11—H11	121.00
C1—C6—C5	119.7 (3)	C12—C13—H13	121.00
Cl1—C7—S1	110.70 (15)	C14—C13—H13	121.00
Cl1—C7—C8	109.6 (2)	C13—C14—H14	120.00
S1—C7—C8	108.8 (2)	C15—C14—H14	120.00
N2—C8—C1	119.6 (3)	C10—C15—H15	120.00
N2—C8—C7	122.2 (3)	C14—C15—H15	120.00
C1—C8—C7	118.2 (3)	N1—C16—H16A	109.00
N3—C9—C10	122.1 (3)	N1—C16—H16B	109.00
C9—C10—C11	118.9 (3)	N1—C16—H16C	109.00
C9—C10—C15	122.0 (4)	H16A—C16—H16B	110.00
C11—C10—C15	119.0 (3)	H16A—C16—H16C	109.00
C10—C11—C12	117.9 (3)	H16B—C16—H16C	109.00
F1—C12—C11	117.2 (4)		
			
O1—S1—N1—C6	56.5 (3)	C2—C1—C8—C7	−169.8 (3)
O2—S1—N1—C6	−170.0 (2)	C8—C1—C2—C3	179.1 (3)
C7—S1—N1—C6	−52.8 (3)	C6—C1—C2—C3	0.4 (5)
O1—S1—N1—C16	−128.2 (3)	C6—C1—C8—N2	−170.9 (3)
O2—S1—N1—C16	5.4 (3)	C1—C2—C3—C4	0.5 (5)
C7—S1—N1—C16	122.6 (3)	C2—C3—C4—C5	−0.6 (5)
N1—S1—C7—Cl1	−63.9 (2)	C3—C4—C5—C6	−0.1 (5)
O1—S1—C7—C8	−58.3 (2)	C4—C5—C6—N1	−178.8 (3)
O2—S1—C7—C8	172.1 (2)	C4—C5—C6—C1	1.0 (5)
N1—S1—C7—C8	56.5 (2)	Cl1—C7—C8—N2	−97.9 (3)
O1—S1—C7—Cl1	−178.68 (18)	Cl1—C7—C8—C1	82.4 (3)
O2—S1—C7—Cl1	51.7 (2)	S1—C7—C8—N2	141.0 (3)
C16—N1—C6—C5	31.8 (5)	S1—C7—C8—C1	−38.7 (3)
S1—N1—C6—C5	−152.9 (3)	N3—C9—C10—C11	178.6 (3)
S1—N1—C6—C1	27.3 (4)	N3—C9—C10—C15	−1.2 (5)
C16—N1—C6—C1	−148.0 (3)	C9—C10—C11—C12	−179.6 (3)
C8—N2—N3—C9	175.3 (3)	C15—C10—C11—C12	0.3 (5)
N3—N2—C8—C1	−179.5 (3)	C9—C10—C15—C14	179.7 (4)
N3—N2—C8—C7	0.9 (4)	C11—C10—C15—C14	−0.1 (5)
N2—N3—C9—C10	179.9 (3)	C10—C11—C12—F1	179.1 (3)
C2—C1—C6—N1	178.7 (3)	C10—C11—C12—C13	−0.3 (6)
C2—C1—C6—C5	−1.1 (4)	F1—C12—C13—C14	−179.3 (3)
C8—C1—C6—N1	0.0 (4)	C11—C12—C13—C14	0.0 (6)
C8—C1—C6—C5	−179.8 (3)	C12—C13—C14—C15	0.2 (6)
C6—C1—C8—C7	8.9 (4)	C13—C14—C15—C10	−0.2 (6)
C2—C1—C8—N2	10.5 (4)		

### Hydrogen-bond geometry (Å, º)

**Table:**

*D*—H···*A*	*D*—H	H···*A*	*D*···*A*	*D*—H···*A*
C5—H5···F1^i^	0.93	2.53	3.442 (5)	167

Symmetry code: (i) *x*, *y*−1, *z*−1.
